# The impact of the war in Ukraine on the perinatal period: Perinatal mental health for refugee women (pmh-rw) protocol

**DOI:** 10.3389/fpsyg.2023.1152478

**Published:** 2023-03-13

**Authors:** Maria F. Rodríguez-Muñoz, Magdalena Chrzan-Dętkoś, Ana Uka, Helena S. García-López, Liudmyla Krupelnytska, Olha Morozova-Larina, Alona Vavilova, Antonina Molotokas, Natalia Murawska, Huynh-Nhu Le

**Affiliations:** ^1^Faculty of Psychology, Universidad Nacional de Educación a Distancia, Madrid, Spain; ^2^Institute of Psychology, University of Gdańsk, Gdańsk, Poland; ^3^Research Center for Sustainable Development and Innovation, Beder University College, Tirana, Albania; ^4^University of Maryland Global Campus (Europe Site), Rota, Spain; ^5^Taras Shevchenko National University of Kyiv, Kyiv, Ukraine; ^6^George Washington University, Washington, DC, United States

**Keywords:** perinatal period, war, anxiety, post-traumatic stress, depression, birth trauma symptoms

## Abstract

**Background:**

The aim of the Perinatal Mental Health for Refugee Women (PMH-RW) Project is to investigate the impact of the war on perinatal mental health: anxiety, post-traumatic stress, depression, and birth trauma symptoms. It will also evaluate the factors that serve as protective elements for the development of these potential diagnoses (such as personality traits, social support, sociodemographic characteristics, and access to medical/mental health services).

**Method:**

An international observational cohort study with baseline data is being assessed in Ukraine (for internal refugees) and several European Countries (for external refugees). The study includes as participants both pregnant women and those who have recently given birth (with babies up to 1 year old). The assessment includes measures on depression (EPDS), anxiety (GAD-7), experiences during birth (City Birth Questionnaire), post-traumatic stress symptoms [Impact of events scale—revised (PTSD-R)], Personality (10-Item Personality Inventory-TIPI), and a questionnaire for socio-demographic data which also such social support.

**Conclusion:**

This study will provide needed information for determining the impact of the Ukrainian Crisis on perinatal mental health by studying potential risk and protective factors. The data collected will be used to inform policymakers with useful information that can be used in the development of plans to protect and promote the mental health of the perinatal refugees impacted by this event. Also, it is our hope that data collected from this study will serve to plant the seeds for further research regarding the impact of the crisis in Ukraine on the offspring and to analyze how these events are affecting further generations.

**Clinical Trial Registration:**

ClinicalTrials.gov, Identifier: NCT05654987.

## Introduction

United Nations Population Fund (UNFPA. Ukraine. Conflict compounds the vulnerabilites of women and girls, 2020), estimated that about 265,000 Ukrainian women were pregnant when the war broke out in February 2022. Eighty thousand women were expected to give birth in the subsequent 3 months in Ukraine or abroad (UNFPA. Ukraine. Conflict compounds the vulnerabilites of women and girls, 2020). The current migratory situation in Ukraine varies. The number of women fleeing the country, returning to Ukraine, and remaining in Ukraine even though the ongoing hostilities is difficult to estimate. However, it can be concluded that many Ukrainian women have experienced and will experience stress and/or possible adverse consequences (or trauma) related to the war and forced migration (Ancheva and Morozova, 2016; Zhabchenko et al., 2018; Romanenko, 2020).

To examine and suggest the most effective practices that may reduce the psychological effects of war refugee status on women, members of the “Research Innovation and Sustainable Pan-European Network in Peripartum Depression Disorder—Riseup-PPD” (Cost Action 18138), funded by the European Cooperation in Science and Technology (COST), established the “Perinatal Mental Health and War Refugees status” group.

Even in non-war conditions, the perinatal period (from pregnancy to the first year after the baby is born) is a vulnerable time *per se*. It is estimated that 1 in 5 women will develop a perinatal mental disorder, which implies that the onset and recurrence of mental health disorders during this period is very high (Andersen et al., 2012; Soto-Balbuena et al., 2018, 2021; Fawcett et al., 2019; Legazpi et al., 2022a,b). Depression, posttraumatic stress disorder (PTSD), and anxiety are shown on the literature as the most recurrent diagnoses (Chrzan-Dętkoś et al., 2022). The consequences of war and migration can be particularly damaging for mothers and babies. Studies have shown that the trauma of war or moving to a new area can have long-term negative effects on mental health, leading to an increased risk of depression and post-traumatic stress disorder (PTSD; Hoppen and Morina, 2019; Hoppen et al., 2021).

Mental health professionals who specialize in working with refugees have identified pregnant women and recent mothers as particularly vulnerable members of the migrant community because they are “in a precarious situation in a foreign country, when the sense of inner homelessness can easily develop, and the capacity for empathy and intuitive parenting can be weakened” (Utari-Witt, Walter, 2021, p. 58). For example, a study carried out in war-affected Syria indicated a high percentage (28.2%) of women is at high risk for postpartum depression (Roumieh et al., 2019).

It is also important to note that experiencing war can increase the chances of having a difficult pregnancy and delivery, as well as negative experiences related to childbirth (Fatusic et al., 2005; Arnetz et al., 2013). Along the same lines, exposure to armed conflict can lead to premature birth and babies with low birth weight (Fatusic et al., 2005; Davis and Sandman, 2010; Keasley et al., 2017). However, the likelihood of premature births, stillbirths, and miscarriages increases when there is direct exposure to conflict. For instance, the negative effects often stem from being exposed to a toxic environment with pollutants, radiation, exhaust fumes, or even water or food that has been contaminated in a war zone (Arnetz et al., 2013). Additionally, emerging research has found that women who fled from the Ukraine war experience significant changes in their status, including a loss of a previous lifestyle, daily contact with friends and relatives, financial losses, changes in perinatal health care associated with limited access to health care, and difficulties in finding a health professional (doctor or midwife) that meets all the medical needs (Chrzan-Dętkoś et al., 2022). Additionally, these women report having limited social support (lack of friends, relatives) and live in isolation as a result of being an immigrant (external refugee); research has found that lack of social support (perceived or actual) is a significant risk factor for developing several mental illnesses during the perinatal period (Marcos-Nájera et al., 2020, 2021).

Another risk factor for mental health is the individual adaptation to a new life in the host country. Heslehurst et al. (2018) reported daily difficulties faced by immigrants and asylum seekers in the host country: not knowing the language and being unfamiliar with the local lifestyle and health practices can create additional burdens and risks for mental and general health. Systematic reviews report that perinatal outcomes such as mental health, maternal mortality, preterm birth and congenital anomalies are predominantly more severe among migrant women (Falah-Hassani et al., 2015: Heslehurst et al., 2018). In a German study, 87.4% of the pre-and postpartum migrants were affected by medical complications or high-risk pregnancies (Kaufmann et al., 2022). However, these studies often do not distinguish among the different statuses of migrant (refugee vs. immigrant) populations, and host countries that may significantly differ regarding the organization of access to support and health care services. For example, an Australian study (Snow et al., 2021) showed elevated psychosocial risk factors among women of refugee background compared with Australia born-women, with the possible under-reporting of mental health problems and family violence. Research on healthcare for pre-, peri- and postpartum refugee women in high-income countries has shown that their access to health care is hampered by immigration experiences, insufficient health literacy, poor language skills, insufficient perception of health needs and use of health services, and legal restrictions (Haith-Cooper and Bradshaw, 2013; Rizkalla et al., 2020). These results raise questions regarding assessing psychosocial risk within different cultural groups and show the importance of the culture-sensitive approach to women with different cultural backgrounds. A systematic review and meta-analysis showed that the prevalence of current PTSD among migrants exposed to armed conflict is 31%, the current major depressive disorder is 25%, and the prevalence of generalized anxiety disorder is 14% (Mesa-Vieira et al., 2022). Migrating to a middle-income or low-income country was associated with an increased prevalence of generalized anxiety disorder—however, evidence is still scarce (Fellmeth et al., 2018).

Pre-migration experiences, migration and experiencing perinatal complications can profoundly impact the lives and mental health of both mother and child and have also been shown to have long-term effects on the mother–child relationship (Graignic-Philippe et al., 2014), day-to-day functioning as well as interpersonal relationships and past traumas (Fellmeth et al., 2018) Protecting and monitoring perinatal mental health in response to the war crisis in Ukraine is crucial (Sacchi, 2022). Focusing on helping this target group of refugees also gives the possibility to reduce the health costs associated with poor perinatal health, such as depression and prevent child development and intergenerational transmission of adversity and trauma. Determining factors that increase the likelihood of developing PPD, anxiety and PTSD in war affected population could help identify women at risk and improve efforts at prevention and early detection. In non-war and forced migration condition, personality traits such as neuroticism (Puyané et al., 2022), perfectionism (Gelabert et al., 2012), poor social support and a personal history of psychopathology (Robertson et al., 2004) are well established risk factors for perinatal mental health disorders—however, not much is known about their role in war affected population.

Generally, research indicates that many factors associated with perinatal depression are more context-specific and vary according to culture (Fellmeth et al., 2018). It is essential to carry out more thorough investigations to evaluate the psychological distress of women, as well as the potential risks and protective factors in varied contexts (internal vs. external refugee) that have emerged due to war, in order to lay a reliable foundation for comprehending the effect of war on women’s mental health during pregnancy.

As Chrzan-Dętkoś et al. (2022) has pointed out, more research should focus on different topics (e.g., risk and protective factors for the perinatal mental health status). The first necessary step should be to develop a comprehensive definition of psychological distress in this target group. Depression and anxiety are the most prevalent psychological diseases in the perinatal period, but PTSD is also an important disorder related to the impact of war and migration (Andersen et al., 2012; Hahn-Holbrook et al., 2017; Shorey et al., 2018; Fawcett et al., 2019). Conducting more research regarding the prevalence and prevention of mental health disorders during the perinatal period is essential. Not only it would benefit the implementation of prevention interventions to support mothers during pregnancy, but also it can have a very positive impact on the offspring (Caparros-Gonzalez et al., 2021). As Tuovinen et al. (2020) state maternal depression and anxiety are strongly related to an increase in inflammatory biomarkers in the mother during pregnancy. These increased numbers of biomarkers are associated with a higher risk of neurodevelopment delay in the offspring. Although fetal programming hypothesis is still under research and results show as inconsistent, there is a valuable truth that can be removed from it: that mothers and babies are interconnected, and that taking care of one implies taking care of the other.

The war in Ukraine brings new challenges to primary care and mental health services all over Europe. We focus on perinatal mental health, and to our knowledge, no previous studies have been conducted to study the impact of war in this field. Although for most European citizens, the date of the war outburst is 24 February 2022, the war in Ukraine, with all the consequences for the civil population, including pregnant and postpartum women, started in 2014. A study conducted in 2016 (Ancheva and Morozova, 2016) demonstrated PTSD frequency in 34.8% of pregnant women displaced internally. Another study showed an increased risk of reactive and personal anxiety, depressive manifestations, autonomic dysfunction, insomnia (Romanenko, 2020), and the risk of premature termination of pregnancy among war-affected population (Cox et al., 1987; Romanenko, 2020). Pregnant women who have been forced to relocate within their own country display a high amount of both reactive and personal anxiety. In pregnant women who were internally displaced, reactive anxiety was 3.3 times higher, and personal anxiety was 2.6 times higher than in pregnant women who were not internally displaced (Zhabchenko et al., 2018). However, to date, there are no publications on changes in the mental health of pregnant women and mothers of newborn children during the full-scale war in Ukraine. The research team intends to launch an international longitudinal study in order to address the lack of knowledge on the consequences of the war and the status of war refugees on maternal mental health during pregnancy. This article outlines the design of the study.

### Aim

The purpose of this upcoming research is to explore how the situation of war in Ukraine may be affecting perinatal mental health, such a anxiety, post-traumatic stress, depression, and birth trauma symptoms.

### Symptoms

Another objective is to evaluate possible risk and protective factors (including social and economic factors, personality traits, social support, and access to medical/mental health services). We are focusing on the two groups of women who were affected by war during pregnancy: those who left Ukraine and are now refugees in other European countries (known as external refugees) and those who chose to remain in Ukraine (in the same place of residence, considered as internal refugees).

The main goals are the following:

1. To evaluate how perinatal mental health has been affected during the war, by observing the differences in (a) levels of depression, anxiety, post-traumatic stress disorder, and birth trauma; as well as in (b) clinical risk indices (proportion of women above the clinical cut-off point on a validated self-report scale)
2. To research which risk and protective factors have a bearing on perinatal mental health during the war.
3. To examine the links between pregnancy results and mental health of women in war-affected areas.
4. To examine the experiences during the birth and delivery, specifically the potential traumatic events during this period.
5. To assess the perinatal women’s needs concerning support and mental health services in Ukraine and in the host countries.

## Methods

### Study design

This is an international observational cohort study. The rationale is to evaluate women’s perinatal mental health experiences throughout pregnancy and the first year after childbirth. The general study will extend from 01 December 2022 to 30 June 2023. This date can be changed depending on the situation of the war.

### Setting

The study will be based online on https://blogs.uned.es/mama/ and carried out in European countries which host war refugees and in Ukraine.

### Participants

The research sample consists of females in the time around childbirth. To be included in the study at the beginning, participants must meet the following criteria:

Being pregnant or a biological mother of an infant up to 12 months of age.
Being 18 years or older.
Being a war refugee from Ukraine (entrance to EU countries from 24 February 2022) or staying in Ukraine after/during the war.
Consenting to take part in the study.

The exclusion criteria are:

Not being currently pregnant or not being the biological mother of an infant up to 12 months of age.
Younger than 18 years of age.
Not consenting to take part in the study.

### Sample size

No restrictions have been put in place for participant registration. Nevertheless, the sample size was determined by taking into account the number of newborns in Ukraine. Consequently, a minimum sample size of 300 individuals will be recruited using a significance level of α-level of 0.05 and variability of 50%. It is essential to bear in mind, though, that the ongoing war situation, which includes the lack of electricity in Ukraine, the possibility of people evacuating cities, and missiles, may make it challenging to reach this sample size.

### Variables and measures

An overview of the assessment measures is provided in Table 1.

**Table 1:** Variables and measures used in the study.

Variables	Measures	References
**Independent/predictor**		
Social support	ACT-PNM	NA
Coping strategies	ACT-PNM	NA
Emotional impact	ACT-PNM	NA
War exposure	ACT-PNM	NA
Pregnancy outcome (birthweight, preterm birth, complications?)	ACT-PNM	NA
Health background/ earlier mental health, substance use,	ACT-PNM	NA
access to the perinatal health services	ACT-PNM	NA
Pregnancy outcome (birthweight, preterm birth, complications?)	ACT-PNM	NA
**Dependent/outcomes**		
Depression symptoms	EPDS	Cox et al. (1987)
Anxiety symptoms	GAD-7	Spitzer et al. (2006)
PTSD symptoms	IES-R	Weiss and Marmar (1997)
Birth PTSD	City BiTS	Ayers et al. (2018)
Personality	The 10-item personality inventory (TIPI)	Gosling et al. (2003)

NA = Not Applicable.

### Assessment, care, and trust—In pregnant and new mothers

This questionnaire includes 40 questions to record sociodemographic data, including current residence, education, number of prior pregnancies, the number of biological children (including miscarriages), the number of individuals residing in their home (adults and children), marital status, whether they are living with a partner, basic information concerning the location after the war outbreak and access to support, changes since the beginning of the war, exposure to armed-conflict or limitations to medical support and social support. This questionnaire was created for this study.

### Edinburgh postnatal depression scale

The Edinburgh Postnatal Depression Scale (EPDS; Cox et al., 1987) is the most widely used self-assessment tool designed to recognized women at risk for perinatal depression (Hahn-Holbrook et al., 2017 < NICE. National Institute for Health and Care Excellence, 2017). This scale includes 10 items measuring common symptoms of depression and anxiety. The scores range from 0 to 30. The cut-off values of 10 or higher and 13 or higher are most often used to identify women who are at risk for depression (Levis et al., 2020). However, 13 has been shown to be the most useful cut-off point established in previous studies. The positive predictive value of EPDS is estimated to be 70% (Cox et al., 1987) or even 90% (Levis et al., 2020). A systematic review has also shown that the EPDS has good psychometric properties in low and middle countries (Shrestha et al., 2016). The EPDS has been validated in over 60 languages (Cox et al., 1987; Gibson et al., 2009; Cox, 2019; Vázquez and Míguez, 2019; Levis et al., 2020). The Ukrainian version, “Единбурзька шкала післяпологової депресії (ЕШПД)” was translated by the study authors.

### Generalized anxiety disorder screener, GAD-7

The GAD-7 (Spitzer et al., 2006) is a 7-item instrument that assesses each of the seven symptoms of general anxiety disorder. The GAD-7 screens for anxiety symptoms in the general population and according to the criteria established by the Diagnostic and Statistical Manual of Mental Disorders (DSM)-IV and DSM-IV-TR (American Psychiatric Association, 1994; American Psychiatric Association, 2000). The GAD-7 total score ranges from 0 to 21. The scale’s total score indicates the level of anxiety symptoms, with higher scores reflecting a greater anxiety severity. Scores of 5, 10, and 15 represent cut-off points for mild, moderate and severe anxiety, respectively (Spitzer et al., 2006). When screening for an anxiety disorder, a recommended cut-off point for referral for further evaluation is 10 or greater (Spitzer et al., 2006). The Ukrainian version of the GAD-7 is available (Romanchuk, 2011) but no psychometric properties have been studied yet. The GAD-7 has shown good psychometric properties during pregnancy (Soto-Balbuena et al., 2021) and the postpartum period (Simpson et al., 2014; Fairbrother et al., 2019).

### Experience during the birth: City BIRTH

The City Birth Questionnaire (Ayers et al., 2018) evaluates PTSD symptoms within the context of childbirth and it contains 29 items related to the DSM-5 diagnostic criteria. It also has two other remaining questions related to the DSM-IV diagnostic criteria. This questionnaire examines participant’s subjective feelings of discomfort related to a specific traumatic event. The City Birth Trauma Scale assesses two factors, Birth-related symptoms and General symptoms of re-experiencing the traumatic event (Ayers et al., 2018; Nakić Radoš et al., 2020). The scores range from 0 to 60. As the authors have pointed out, the scores for percentiles are 25th = 3, 50th = 9, 75th = 18. The questionnaire has shown good psychometric properties (Ayers et al., 2018; Nakić Radoš et al., 2020).

The Ukrainian version, “Міська шкала пологової травми” was translated for the purpose of the study by the authors.

### Impact of events scale-revised

The Impact of Event Scale-Revised (IES-R; Weiss and Marmar, 1997) is a self-assessment tool measure for capturing the level of symptomatic response to specific traumatic stressors as it was manifested in the previous 7 days.

The scale has been translated into different languages (Baguena et al., 2001; Asukai et al., 2002) and different samples (Beck et al., 2008) with good psychometric properties.

A revised version of the Impact of Event Scale-Revised by Weiss and Marmar (1997), contains 22 items with scores gathered using a 5-point scale (0–4). What differentiates this revised version of the Impact of Event Scale (IES-R) from the previous one is that this latest version has seven additional questions and a scoring range of 0 to 88.

On this test, a score of 24 or more is clinically significant as it is indicative of an individual having full or partial PTSD. A score of 33 or more is the most accurate way to determine the probability of PTSD, and a score of 37 or higher is a strong indication of the disorder (Weiss, 2007).

This questionnaire considers the three dimensions of PTSD according to DSM-IV (Weiss and Marmar, 1997), including the following: Intruding recurring images, dreams, and/or thoughts or perceptual impressions related to the traumatic event; Agitation characterized by increased alertness, anxiety, impatience, and/or difficulty concentrating attention; and Avoidance manifested by efforts to get rid of thoughts, emotions, or conversations related to the traumatic event.

The Ukrainian version “Шкала впливу стресових подій – переглянута (ШВСП-П)” was translated into Ukrainian by the study authors.

### Ten-item personality inventory

The Ten-Item Personality Inventory (TIPI; Gosling et al., 2003) measures the “Big Five” traits of personality, including neuroticism, extraversion, conscientiousness, openness to experience, and agreeableness (Costa and McCrae, 1992). Research shows that this two-minute questionnaire is a relatively accurate and reliable tool for measuring personality (Sosnowska et al., 2020). In each of the 5 subscales, scores range from 2 to 14 (two points on each subscale; the scores range for each point is from 1 to 7) TIPI scale scoring (“R” denotes reverse-scored items): Extraversion: 1, 6R; Agreeableness: 2R, 7; Conscientiousness; 3, 8R; Emotional Stability: 4R, 9; Openness to Experiences: 5, 10R (Gosling et al., 2003). This questionnaire has a Ukranian adaptation (Klimanska and Haletska, 2019).

### Translations of the measures

Three instruments were translated to Ukrainian in this study. The Edinburgh Postnatal Depression Scale (Cox et al., 1987) was translated by L.K., O. M-L., and A.V. The City Birth Trauma Scale (Ayers et al., 2018) was translated by L.K., O. M-L., A.V., and V.S. The impact of events scale-revised (Weiss and Marmar’s, 1997) was translated by L.K., O. M-L., and V.K. The translation was carried out according to Brislin’s translation model (Brislin, 1970; 1986), applicable to the preparation of instruments for cross-cultural research. The back translations were carried out, and the detailed procedure of the translations is available from the authors.

### Ethical standards

This study has been conducted in accordance with the protocols stated in the Declaration of Helsinki. Ethical authorization was given (21-PSI-2022). All national guidelines for data security are followed in the management of study data. Digitalized informed consent will be received from all members, and the confidentiality of all the data will be protected. The study design has been preregistered in ClinicalTrials.gov (ref: NCT05654987) following gold standards in research on cross-sectional studies, STROBE (Vandenbroucke et al., 2007).

Participation in the study can elicit changes in one’s emotional state. To address this specific problem, at the end of the survey, a list of up-to-date services and resources will be provided where the participants can obtain information and help concerning mental health services and lactation advice. In addition, contact details of the lead research team for each nation will be available if participants wish to seek out further information. On request, the researchers will provide the participants with the global results of the study.

### Procedure

Potential participants will be sought out through social media platforms (e.g., Twitter, Facebook, Instagram, LinkedIn, ResearchGate, WhatsApp), networks of educational institutions, healthcare facilities, and non-governmental organizations working in the area of perinatal mental health, policymakers, local organizations, and other stakeholders (utilizing the contacts provided by the Riseup-PPD), in addition to the personal networks of the research team members and through direct contact *via* text or email.

Participants taking part in the study will be asked to click on the website link found on the general project website^1^ in order to access the online questionnaire. They will then be presented with an electronic consent form that outlines the purpose and content of the questions, the risks and benefits, and the ethical considerations (such as voluntary participation, data confidentiality, secure storage of data, and the lack of financial compensation). At the bottom of the form, they must confirm that they meet the eligibility criteria and provide consent for the study. If a participant does not meet the criteria, they will be directed to a message thanking them for their interest and informing them of the requirements for participation. Completion of the baseline questionnaire is estimated to take around 35 min.

### Data analysis

#### Descriptive

At the beginning, descriptive statistics will be examined for all the study variables. To analyze the presence or not of the different variables (depression, anxiety or PTSD) concerning the different categorical variables, contingency tables will be made using *Pearson’s Chi-Square*. *Cramer’s V* will be used to calculate the effect size. Likewise, when the variables are continuous, Student’s *t* will be used, and the effect size will be calculated using Cohen’s *d.*

#### Psychometric properties

To examine the psychometric properties of the questionnaires in the Ukrainian language, several analyses will be reported. Exploratory Factor Analyses (EFA) will be conducted using Statistical Package for the Social Sciences (SPSS), Version 26 software. Analysis of the factor dimensional constraints will determine each model’s underlying individual item loadings. Based on the results of the EFA, Confirmatory Factor Analysis (CFA) will then be analyzed. ECA will be used to analyze how to fit the models using several indexes such as Satorra-Bentler χ^2^, CFI, RMSEA and the confidence interval of RMSEA statistics will be calculated. Lagrange multipliers and Wald test will be sequentially performed to improve the models’ fitness, following the study’s hypotheses. Standardized coefficients and measurement equations with scores in R2 will be reported. These analyses will be conducted using the AMOS program.

Prediction models. To examine the risk and protective factors associated with perinatal mental health outcomes, a random sample of 70% of participants will be used to derive the machine learning algorithms, including regularized logistic regression, random forest, decision tree, and gradient boosting to predict the risk of perinatal mental health outcomes, and their performance, and the remaining 30% for validation. First, in the derivation sample, all predictors described above will be included in the models to estimate the probability of depression, anxiety, or PTSD. Beginning with a model containing all potential covariates, the variable with the least significant *p* value will be removed and tested using the likelihood-ratio test until all variables left in the model significantly (at alpha = 0.05) contributed to the model.

In regularized logistic regression models, results will be presented as Odds Ratio (OR) with 95% confidence intervals (CIs).

Logistic regression analyses will be conducted using the steps forward procedure to examine which variables will predict the study’s dependent variables. This regression analysis makes it possible to predict a dichotomous dependent variable based on predictor, categorical or quantitative variables the R^2^ will be used to calculate the proportion of the explained variance of clinical outcomes by the selected predictors. The different aspects of model performance will be studied. The receiver operating characteristics (ROC) curves (and the corresponding area under the ROC curve—AUC) will also be calculated to test for discrimination characteristics.

### Dissemination and data sharing plan

Data and resources obtained and utilized for the study will be made available to other qualified researchers in accordance with academic standards. Those who wish to gain access to the datasets and analyses can do so upon making a legitimate request. The results of the study will be published in peer-reviewed publications and presented at scholarly conferences both domestically and abroad.

## Discussion

The current war in Ukraine affects every other European country in some ways. Since the war outbreak, the neighboring countries have absorbed and registered a high number of refugees on a daily basis (Chrzan-Dętkoś et al., 2022; Kumar et al., 2022; Lloyd and Sirkeci, 2022). Although the number of war refugees varies in European countries, every country faces similar challenges in providing adequate support and possible medical and psychological interventions for women during this vulnerable period; these challenges include language barriers, provision of culturally sensitive services and trauma-informed care, and equitable access to health and mental health services in each European country. Our study aims to assess the mental health needs of perinatal Ukrainian women in their home country and abroad to ascertain the risk and protective factors and the provision of services needed during the perinatal period. Systematic reviews (Hajak et al., 2021) show that migrants who have experienced armed conflict are particularly vulnerable to mental health issues and require mental health assistance. Women in the perinatal period are a vulnerable group, and their mental health may be further exacerbated during wartime.

The midwifery and obstetric care systems in European countries may be an important reference in identifying and caring for those affected. Screening for depression and anxiety with the EPDS and GAD-7, for example, may reduce the disparities observed in the cited literature. Knowing the rate and severity of mental health symptoms and the needs of female war refugees is the first step to creating a plan for helping and preparing services to supply this group of women.

Based on the literature, interventions supporting war-affected mothers and mother—babies dyads should be comprehensive, sustainable, and devoid of harm. Support should be multi-level, resilience-oriented, multidisciplinary, and tailored to the needs of the individual mother (Tol et al., 2011). The Inter-Agency Standing Committee (2007) proposed a multilevel intervention pyramid for mental health and psychosocial support in emergencies, which includes four levels of intervention: (1) provision of basic services and security, (2) community and family support, (3) focused non-specialized support, and (4) specialized support. All layers of the pyramid are essential and should be implemented concurrently according to the needs of the individual. However, to implement it, we need to begin to understand the impact of perinatal mental health of Ukranian perinatal women by investigate prevalence and associated risk and protective factors of mental health problems and the special needs of this target group.

Research shows that many of the European countries do not report specific clinical recommendations or specific guidelines for managing peripartum depression even for women living under normal conditions. Additionally, the existing guidelines vary a lot in the number and themes of clinical recommendations (Motrico et al., 2022). Therefore, the results from this study could assist The European Union and other nations’ policymakers to Create a set of rules and strategies to help reduce the occurrence of mental health issues during pregnancy and childbirth in the Ukrainian crisis and other potential armed conflicts and war-forced migration to EU countries. The United Nations High Commissioner for Refugees (2019) report shows that due to persecution, armed conflicts, deficient healthcare and human rights violations, more than 70 million people worldwide were forcibly displaced from their homes by the end of 2018, with approximately one-sixth seeking protection abroad. Women account for nearly 50% of all refugees, internally displaced, and stateless persons (United Nations High Commissioner for Refugees, 2019). In the literature, we can find interesting reflections focusing on the methodology of studies of immigrants’ mental health. For example, Crumlish and O’Rourke (2010) observed that very few scientists from refugee communities lead research in their communities or the refugee mental health field in general. Planning and implementation of research aimed at understanding these survivors are mainly in the hands of “mainstream academia.” In our study, we could reduce this bias while working in the international team and applying the study protocol together with Ukrainian researchers.

## Conclusion

To sum up, this study is an international research project created to evaluate the effect of the Ukrainian War on the mental health of women during pregnancy. It will allow for a thorough overview of how the war has affected mental wellbeing during the perinatal period, which could then be used to support and guide refugees and providers in different situations. Ultimately, this research should provide concrete data for policymakers and healthcare professionals to utilize.

## Ethics statement

The studies involving human participants were reviewed and approved by Comité de Ética de la Investigación, UNED (Madrid), the Research Ethics Committee of the Faculty of Psychology of Taras Shevchenko National University of Kyiv. The patients/participants provided their written informed consent to participate in this study.

## Author contributions

MR-M contributed to conception and design of the study and wrote the first draft of the manuscript. MC-D contributed to conception and design of the study. AU contributed to conception. HG-L organized the database and wrote the first draft. LK and OM-L contributed to conception and design of the study and provided psychometric adaptation. AV provided psychometric adaptation and prepared collecting data. AM prepared collecting data. NM contributed to design of the study. H-NL reviewed and edit the final version. All authors contributed to the article and approved the submitted version.

## Funding

This paper is based upon work from the COST Action Riseup-PPD CA 18138 and was supported by COST under COST Action Riseup-PPD CA18138 www.cost.eu.

## Conflict of interest

The authors declare that the research was conducted in the absence of any commercial or financial relationships that could be construed as a potential conflict of interest.

## Publisher’s note

All claims expressed in this article are solely those of the authors and do not necessarily represent those of their affiliated organizations, or those of the publisher, the editors and the reviewers. Any product that may be evaluated in this article, or claim that may be made by its manufacturer, is not guaranteed or endorsed by the publisher.

## Footnotes

^1^ https://www.blog.une.es.mama

## References

[ref1] American Psychiatric Association (1994). Diagnostic and statistical manual of mental disorders. 4th Edn.

[ref2] American Psychiatric Association. (2000). Diagnostic and statistical manual of mental disorders. (4th) Edn.

[ref4] Ancheva I. Morozova K. (2016). Post-traumatic stress disorder amongst internally displaced pregnant females. Available at: https://repo.odmu.edu.ua:443/xmlui/handle/123456789/7411

[ref5] Andersen L. B. Melvaer L. B. Videbech P. Lamont R. F. Joergensen J. S. (2012). Risk factors for developing post-traumatic stress disorder following childbirth: a systematic review. Acta Obstet. Gynecol. Scand. 91, 1261–1272. doi: 10.1111/j.1600-0412.2012.01476.x, PMID: 22670573

[ref6] Arnetz J. Rofa Y. Arnetz B. Ventimiglia M. Jamil H. (2013). Resilience as a protective factor against the development of psychopathology among refugees. J. Nerv. Ment. Dis., 201, 167–172. doi: 10.1097/NMD.0b013e3182848afe PMC3584239 23407208

[ref7] Asukai N. Kato H. Kawamura N. Kim Y. Yamamoto K. Kishimoto J.. (2002). Reliability and validity of the Japanese-language version of the impact of event scale-revised (IES-R-J): four studies of different traumatic events. J. Nerv. Ment. Dis. 190, 175–182. doi: 10.1097/00005053-200203000-00006, PMID: 11923652

[ref8] Ayers S. Wright D. B. Thornton A. (2018). Development of a measure of postpartum PTSD: the City birth trauma scale. Front. Psych. 9. doi: 10.3389/fpsyt.2018.00409, PMID: 30279664 PMC6153962

[ref9] Baguena M. Villarroya E. Beleña M. Díaz Martinez A. Roldan C. Reig R. (2001). Psychometric properties of the Spanish version of the impact of event scale-revised (IES-R). Analisis y Modificacion de Conducta 27, 581–604.

[ref10] Beck J. G. Grant D. M. Read J. P. Clapp J. D. Coffey S. F. Miller L. M.. (2008). The impact of event scale –revised: psychometric properties in a sample of motor vehicle accident survivors. J. Anxiety Disord. 22, 187–198. doi: 10.1016/j.janxdis.2007.02.007, PMID: 17369016 PMC2259224

[ref001] Brislin R. W. (1970). Back-translation for cross-cultural research. J. Cross. Cult. Psychol. 1, 185–216. doi: 10.1177/135910457000100301

[ref002] Brislin R. W. (1986). “The wording and translation of research instruments,” in Field methods in cross-cultural research. (Sage Publications, Inc.), 137–164.

[ref11] Caparros-Gonzalez R. A. Romero-Gonzalez B. Puertas-Gonzalez J. A. Quirós-Fernández S. Coca-Guzmán B. Peralta-Ramirez M. I. (2021). Matronas y profesionales de Psicología ante el screening y prevención de estrés específico del embarazo [Midwives and psychologists as profesionals to screen and prevent pregnancy-specific stress.]. Rev. Esp. Salud Publica 95:e202104060 33896933

[ref12] Chrzan-Dętkoś M. Rodríguez-Muñoz M. F. Krupelnytska L. Morozova-Larina O. Vavilova A. López H. G.. (2022). Good practices in perinatal mental health for women during wars and migrations: a narrative synthesis from the COST action Riseup-PPD in the context of the war in Ukraine. Clinica y Salud 33, 127–135. doi: 10.5093/clysa2022a14

[ref003] Costa P. T. McCrae R. R. (1992). The five-factor model of personality and its relevance to personality disorders. J. Personal. Disord. 6, 343–359. doi: 10.1521/pedi.1992.6.4.343

[ref13] Cox J. (2019). Thirty years with the Edinburgh postnatal depression scale: voices from the past and recommendations for the future. Br. J. Psychiatry 214, 127–129. doi: 10.1192/bjp.2018.245, PMID: 30774059

[ref14] Cox J. L. Holden J. M. Sagovsky R. (1987). Detection of postnatal depression. Development of the 10-item Edinburgh postnatal depression scale. Br. J. Psychiatry J. Ment. Sci. 150, 782–786. doi: 10.1192/bjp.150.6.782 3651732

[ref15] Crumlish N. O’Rourke K. (2010). A systematic review of treatments for post-traumatic stress disorder among refugees and asylum-seekers. J. Nerv. Ment. Dis. 198, 237–251. doi: 10.1097/NMD.0b013e3181d61258 20386252

[ref16] Davis E. P. Sandman C. A. (2010). The timing of prenatal exposure to maternal cortisol and psychosocial stress is associated with human infant cognitive development. Child Dev. 81, 131–148. doi: 10.1111/j.1467-8624.2009.01385.x, PMID: 20331658 PMC2846100

[ref17] Fairbrother N. Corbyn B. Thordarson D. S. Ma A. Surm D. (2019). Screening for perinatal anxiety disorders: room to grow. J. Affect. Disord. 250, 363–370. doi: 10.1016/j.jad.2019.03.052, PMID: 30877859

[ref18] Falah-Hassani K. Shiri R. Vigod S. Dennis C.-L. (2015). Prevalence of postpartum depression among immigrant women: a systematic review and meta-analysis. J. Psychiatr. Res. 70, 67–82. doi: 10.1016/j.jpsychires.2015.08.010, PMID: 26424425

[ref19] Fatusic Z. Kurjak A. Grgic G. Tulumovic A. (2005). The influence of the war on perinatal and maternal mortality in Bosnia and Herzegovina. J. Matern. Fetal Neonatal Med. 18, 259–263. doi: 10.1080/147670500198501, PMID: 16318977

[ref20] Fawcett E. J. Fairbrother N. Cox M. L. White I. R. Fawcett J. M. (2019). The prevalence of anxiety disorders during pregnancy and the postpartum period: a multivariate Bayesian meta-analysis. J. Clin. Psychiatry 80:18r12527. doi: 10.4088/JCP.18r12527, PMID: 31347796 PMC6839961

[ref21] Fellmeth G. Plugge E. H. Nosten S. Oo M. M. Fazel M. Charunwatthana P.. (2018). Living with severe perinatal depression: a qualitative study of the experiences of labour migrant and refugee women on the Thai-Myanmar border. BMC Psychiatry 18:229. doi: 10.1186/s12888-018-1815-7, PMID: 30012124 PMC6048862

[ref22] Gelabert E. Subirà S. García-Esteve L. Navarro P. Plaza A. Cuyàs E.. (2012). Perfectionism dimensions in major postpartum depression. J. Affect. Disord. 136, 17–25. doi: 10.1016/j.jad.2011.08.030, PMID: 21930303

[ref23] Gibson J. McKenzie-McHarg K. Shakespeare J. Price J. Gray R. (2009). A systematic review of studies validating the Edinburgh postnatal depression scale in antepartum and postpartum women. Acta Psychiatr. Scand. 119, 350–364. doi: 10.1111/j.1600-0447.2009.01363.x, PMID: 19298573

[ref24] Gosling S. D. Rentfrow P. J. Swann W. B. Jr. (2003). A very brief measure of the big-five personality domains. J. Res. Pers. 37, 504–528. doi: 10.1016/S0092-6566(03)00046-1

[ref25] Graignic-Philippe R. Dayan J. Chokron S. Jacquet A.-Y. Tordjman S. (2014). Effects of prenatal stress on fetal and child development: a critical literature review. Neurosci. Biobehav. Rev. 43, 137–162. doi: 10.1016/j.neubiorev.2014.03.022, PMID: 24747487

[ref26] Hahn-Holbrook J. Cornwell-Hinrichs T. Anaya I. (2017). Economic and health predictors of National Postpartum Depression Prevalence: a systematic review, meta-analysis, and meta-regression of 291 studies from 56 countries. Front. Psych. 8:248. doi: 10.3389/fpsyt.2017.00248, PMID: 29449816 PMC5799244

[ref27] Haith-Cooper M. Bradshaw G. (2013). Meeting the health and social care needs of pregnant asylum seekers; midwifery students’ perspectives: part 3; «the pregnant woman within the global context»; an inclusive model for midwifery education to address the needs of asylum seeking women in the UK. Nurse Educ. Today 33, 1045–1050. doi: 10.1016/j.nedt.2012.04.016, PMID: 22647390

[ref004] Hajak V. L. Sardana S. Verdeli H. Grimm N. (2021). A systematic review of factors affecting mental health and well-being of asylum seekers and refugees in Germany. Front. Psy. 12:643704. doi: 10.3389/fpsyt.2021.643704 PMC8012840 33815176

[ref28] Heslehurst N. Brown H. Pemu A. Coleman H. Rankin J. (2018). Perinatal health outcomes and care among asylum seekers and refugees: a systematic review of systematic reviews. BMC Med. 16:89. doi: 10.1186/s12916-018-1064-0, PMID: 29890984 PMC5996508

[ref29] Hoppen T. H. Morina N. (2019). The prevalence of PTSD and major depression in the global population of adult war survivors: a meta-analytically informed estimate in absolute numbers. Eur. J. Psychotraumatol. 10:1578637. doi: 10.1080/20008198.2019.1578637, PMID: 30834069 PMC6394282

[ref30] Hoppen T. H. Priebe S. Vetter I. Morina N. (2021). Global burden of post-traumatic stress disorder and major depression in countries affected by the war between 1989 and 2019: a systematic review and meta-analysis. BMJ Glob. Health 6:e006303. doi: 10.1136/bmjgh-2021-006303, PMID: 34321235 PMC8319986

[ref31] Inter-Agency Standing Committee (2007). IASC guidelines on mental health and psychosocial support in emergency settings. Geneva: IASC. 10.1080/09540261.2022.2147420 36502397

[ref32] Kaufmann C. Zehetmair C. Jahn R. Marungu R. Cranz A. Kindermann D.. (2022). Maternal mental healthcare needs of refugee women in a state registration and reception Centre in Germany: a descriptive study. Health Soc. Care Community 30, 1608–1617. doi: 10.1111/hsc.13508, PMID: 34250665

[ref33] Keasley J. Blickwedel J. Quenby S. (2017). Adverse effects of exposure to armed conflict on pregnancy: a systematic review. BMJ Glob. Health 2:e000377. doi: 10.1136/bmjgh-2017-000377, PMID: 29333283 PMC5706483

[ref34] Klimanska M. Haletska I. (2019). Ukrainian adaptation of the short five factor personality questionnaire TIPI (TIPI-UKR). Psychol. J. 5, 57–74. doi: 10.31108/1.2019.5.9.4

[ref35] Kumar B. N. James R. Hargreaves S. Bozorgmehr K. Mosca D. Hosseinalipour S. M.. (2022). Meeting the health needs of displaced people fleeing Ukraine: drawing on existing technical guidance and evidence. Lancet Reg. Health Eur. 17:100403. doi: 10.1016/j.lanepe.2022.100403 35721694 PMC9198835

[ref36] Legazpi P. C. C. Rodríguez-Muñoz M. F. Le H.-N. Balbuena C. S. Olivares M. E. Méndez N. I. (2022a). Suicidal ideation: prevalence and risk factors during pregnancy. Midwifery 106:103226. doi: 10.1016/j.midw.2021.103226, PMID: 34990995

[ref37] Legazpi P. C. C. Rodríguez-Muñoz M. F. Olivares-Crespo M. E. Izquierdo-Méndez N. (2022b). Review of suicidal ideation during pregnancy: risk factors, prevalence, assessment instruments and consequences. Psicologia Reflexão e Crítica 35:13. doi: 10.1186/s41155-022-00220-4, PMID: 35606474 PMC9127017

[ref38] Levis B. Negeri Z. Sun Y. Benedetti A. Thombs B. D. (2020). Accuracy of the Edinburgh postnatal depression scale (EPDS) for screening to detect major depression among pregnant and postpartum women: systematic review and meta-analysis of individual participant data. BMJ 371:m4022. doi: 10.1136/bmj.m4022, PMID: 33177069 PMC7656313

[ref39] Lloyd A. T. Sirkeci I. (2022). A long-term view of refugee flows from Ukraine: war, insecurities, and migration. Migr. Lett. 19, 523–535. doi: 10.33182/ml.v19i4.2313

[ref40] Marcos-Nájera R. Rodríguez-Muñoz M. F. Lara M. A. Navarrete L. Le H.-N. (2021). A cross-cultural analysis of the prevalence and risk factors for prenatal depression in Spain and Mexico. Cult. Med. Psychiatry 45, 599–612. doi: 10.1007/s11013-020-09691-5, PMID: 33098543

[ref41] Marcos-Nájera R. Rodríguez-Muñoz M. d. l. F. Soto Balbuena C. Olivares Crespo M. E. Izquierdo Méndez N. Le H.-N.. (2020). The prevalence and risk factors for antenatal depression among pregnant immigrant and native women in Spain. J. Transcult. Nurs. 31, 564–575. doi: 10.1177/1043659619891234, PMID: 31779531

[ref42] Mesa-Vieira C. Haas A. D. Buitrago-Garcia D. Roa-Diaz Z. M. Minder B. Gamba M.. (2022). Mental health of migrants with pre-migration exposure to armed conflict: a systematic review and meta-analysis. Lancet Public Health 7, e469–e481. doi: 10.1016/S2468-2667(22)00061-5, PMID: 35487232

[ref43] Motrico E. Moreno-Peral P. Uriko K. Hancheva C. Brekalo M. Ajaz E.. (2022). Clinical practice guidelines with recommendations for peripartum depression: a European systematic review. Acta Psychiatr. Scand. 146, 325–339. doi: 10.1111/acps.13478, PMID: 35838293 PMC9805017

[ref44] Nakić Radoš S. Matijaš M. Kuhar L. Anđelinović M. Ayers S. (2020). Measuring and conceptualizing PTSD following childbirth: validation of the City birth trauma scale. Psychol. Trauma Theory Res. Pract. Policy 12, 147–155. doi: 10.1037/tra0000501, PMID: 31368743

[ref45] NICE. National Institute for Health and Care Excellence (2017). Antenatal and postnatal mental health: Clinical management and service guidance. British Psychological Society. Retrieved from https://www.nice.org.uk/guidance/cg192 31990493

[ref47] Puyané M. Subirà S. Torres A. Roca A. Garcia-Esteve L. Gelabert E. (2022). Personality traits as a risk factor for postpartum depression: a systematic review and meta-analysis. J. Affect. Disord. 298, 577–589. doi: 10.1016/j.jad.2021.11.010, PMID: 34763034

[ref48] Rizkalla K. Maar M. Pilon R. McGregor L. Reade M. (2020). Improving the response of primary care providers to rural first nation women who experience intimate partner violence: a qualitative study. BMC Womens Health 20:209. doi: 10.1186/s12905-020-01053-y, PMID: 32957935 PMC7507614

[ref49] Robertson E. Grace S. Wallington T. Stewart D. E. (2004). Antenatal risk factors for postpartum depression: a synthesis of recent literature. Gen. Hosp. Psychiatry 26, 289–295. doi: 10.1016/j.genhosppsych.2004.02.006, PMID: 15234824

[ref50] Romanchuk O. (2011). Опитувальник з генералізованої тривоги. Київський інститут когнітивно-біхевіоральної терапії. Available at: https://i-cbt.org.ua

[ref51] Romanenko I. Y. (2020). Динамика показателей психоэмоционального состояния женщин—Внутренне перемещенных лиц с угрозой прерывания беременности на фоне комплексного лечения. Int. J. Endocrinol. 16, 686–691. doi: 10.22141/2224-0721.16.8.2020.222890

[ref52] Roumieh M. Bashour H. Kharouf M. Chaikha S. (2019). Prevalence and risk factors for postpartum depression among women seen at primary health care Centres in Damascus. BMC Pregnancy Childbirth 19:519. doi: 10.1186/s12884-019-2685-9, PMID: 31870326 PMC6929307

[ref53] Sacchi C. (2022). Protection of perinatal mental health during the war in Ukraine. Lancet Reg. Health Eur 15:100362. doi: 10.1016/j.lanepe.2022.100362, PMID: 35531497 PMC9072996

[ref54] Shorey S. Chee C. Y. I. Ng E. D. Chan Y. H. Tam W. W. S. Chong Y. S. (2018). Prevalence and incidence of postpartum depression among healthy mothers: a systematic review and meta-analysis. J. Psychiatr. Res. 104, 235–248. doi: 10.1016/j.jpsychires.2018.08.001, PMID: 30114665

[ref55] Shrestha S. D. Pradhan R. Tran T. D. Gualano R. C. Fisher J. R. (2016). Reliability and validity of the Edinburgh postnatal depression scale (EPDS) for detecting perinatal common mental disorders (PCMDs) among women in low-and lower-middle-income countries: a systematic review. BMC Pregnancy Childbirth 16:72. doi: 10.1186/s12884-016-0859-2, PMID: 27044437 PMC4820998

[ref56] Simpson W. Glazer M. Michalski N. Steiner M. Frey B. N. (2014). Comparative efficacy of the generalized anxiety disorder 7-item scale and the Edinburgh postnatal depression scale as screening tools for generalized anxiety disorder in pregnancy and the postpartum period. Can. J. Psychiatry 59, 434–440. doi: 10.1177/070674371405900806, PMID: 25161068 PMC4143300

[ref57] Snow G. Melvin G. A. Boyle J. A. Gibson-Helm M. East C. E. McBride J.. (2021). Perinatal psychosocial assessment of women of refugee background. Women Birth 34, e302–e308. doi: 10.1016/j.wombi.2020.05.009, PMID: 32571715

[ref005] Sosnowska J. Kuppens P. De Fruyt T. Hofmans J. (2020). New directions in the conceptualization and assessment of personality—A dynamic systems approach. Eur. J. Pers. 34, 988–998. doi: 10.1002/per.2233, PMID: 15234824

[ref58] Soto-Balbuena C. Rodriguez-Muñoz M. F. Escudero A. Ferrer F. J. Grupo P. M. B. H. (2018). Incidence, prevalence and risk factors related to anxiety symptoms during pregnancy. Psicothema 30, 257–263. doi: 10.7334/psicothema2017.379 30009746

[ref59] Soto-Balbuena C. Rodríguez-Muñoz M. F. Le H.-N. (2021). Validation of the generalized anxiety disorder screener (GAD-7) in Spanish pregnant women. Psicothema 33, 164–170. doi: 10.7334/psicothema2020.167, PMID: 33453750

[ref60] Spitzer R. L. Kroenke K. Williams J. B. W. Löwe B. (2006). A brief measure for assessing generalized anxiety disorder: the GAD-7. Arch. Intern. Med. 166, 1092–1097. doi: 10.1001/archinte.166.10.1092 16717171

[ref61] Tol W. A. Barbui C. Galappatti A. Silove D. Betancourt T. S. Souza R.. (2011). Mental health and psychosocial support in humanitarian settings: linking practice and research. Lancet 378, 1581–1591. doi: 10.1016/S0140-6736(11)61094-5, PMID: 22008428 PMC3985411

[ref62] Tuovinen S. Lahti-Pulkkinen M. Rantalainen V. Kajantie E. Räikkönen K. (2020). Prenatal programming of child neurocognitive abilities and maternal mental health. Curr. Opin. Endocr. Metab. Res. 13, 28–38. doi: 10.1016/j.coemr.2020.09.001

[ref64] UNFPA. Ukraine. Conflict compounds the vulnerabilites of women and girls (2020). Appeal for Ukraine. Available at: https://www.unfpa.org/resources/unfpa-appeal-ukraine

[ref65] United Nations High Commissioner for Refugees. (2019). Global Trends. Forced Displacement in 2018. Available at: https://www.unhcr.org/statistics/unhcrstats/5d08d7ee7/unhcr-global-trends-2018.html.

[ref66] Utari-Witt H. Walter A. (2021) W drodze na obca ziemię. Psychoanalityczne refleksje na temat migracji. (On the way to a foreign land. Psychoanalytic Reflections on Migration.) Warszawa: Waszawskie Centrum Innowacji Edukacyjno – Społecznych i Szkoleń.

[ref67] Vandenbroucke J. P. von Elm E. Altman D. G. Gøtzsche P. C. Mulrow C. D. Pocock S. J.. (2007). Strengthening the reporting of observational studies in epidemiology (STROBE): explanation and elaboration. PLoS Med. 4:e297. doi: 10.1371/journal.pmed.0040297, PMID: 17941715 PMC2020496

[ref68] Vázquez M. B. Míguez M. C. (2019). Validation of the Edinburgh postnatal depression scale as a screening tool for depression in Spanish pregnant women. J. Affect. Disord. 246, 515–521. doi: 10.1016/j.jad.2018.12.075, PMID: 30599376

[ref69] Weiss D. S. (2007). “The impact of event scale-revised” in Assessing psychological trauma and PTSD: A practitioner's handbook. eds. Wilson J. P. Keane T. M.. 2nd ed (New York: Guilford Press), 168–189.

[ref70] Weiss D. S. Marmar C. R. (1997). “The impact of event scale—revised” in En *assessing psychological trauma and PTSD*. eds. Wilson J. P. Keane T. M.. (The Guilford Press), 399–411.

[ref74] Zhabchenko I. A. Kornietz N. G. Tertychnaya-Telyuk S. V. Kovalenko T. N. (2018). Peculiarities of psychoemotional condition of pregnant women-displaced persons. Rep. Vinnyt. Natl. Med. Univ. 22, 99–103. doi: 10.31393/reports-vnmedical-2018-22(1)-19

